# Age-dependent changes in rat lacrimal gland anti-oxidant and vesicular related protein expression profiles

**Published:** 2012-01-25

**Authors:** Thiago Martins Batista, Lilian Midori Tomiyoshi, Ana Carolina Dias, Letícia Prates Roma, Carolina Maria Módulo, Leonardo Tannus Malki, Elísio Bueno Machado Filho, Rafael Deminice, Alceu Afonso Jordão, Daniel A. Cunha, Eduardo Melani Rocha

**Affiliations:** 1 Department of Physiology, Institute of Biology, Unicamp, Campinas, Brazil; 2 Department of Ophthalmology, Otorhinolaryngology and Head & Neck Surgery, Faculty of Medicine of Ribeirão Preto, São Paulo University (USP), Ribeirão Preto, SP, Brazil; 3 Department of Clinical Medicine, Faculty of Medicine of Ribeirão Preto, São Paulo University (USP), Ribeirão Preto, SP, Brazil

## Abstract

**Purpose:**

Anti-oxidation and exocytosis are important for maintaining exocrine tissue homeostasis. During aging, functional and structural alterations occur in the lacrimal gland (LG), including oxidative damage to proteins, lipids, and DNA. The aims of the present study were to determine in the aging LG: a) the effects of aging on LG structure and secretory activity and b) changes in the expression of oxidative stress markers.

**Methods:**

To address these goals, tear secretion composition and corneal impression cytology were compared between male Wistar rats of 2 (control) and 24 (aged) months. LG morphology and the expression levels of vitamin E and malonaldehyde (MDA) were evaluated to determine the anti-oxidant activity and lipid peroxidation, respectively. RT–PCR and western blot analysis were used for the analysis of Ras related in brain GTPase protein (Rab) and soluble N-ethylmaleimide-sensitive factor attachment protein receptor (SNARE) proteins of the secretory machinery (i.e.; Rab 3d, Rab 27, vesicle-associated membrane protein-2 (Vamp-2), and syntaxin).

**Results:**

Histological analysis of aged rats revealed a higher frequency of corneal epithelia metaplasia. In the acinar cells, organelles underwent degeneration, and lipofucsin-like material accumulated in the cytoplasm along with declines in the anti-oxidant marker vitamin E. *Rab3d* and *Rab27b* mRNA levels fell along with Rab3d protein expression, whereas syntaxin levels increased.

**Conclusions:**

These findings indicate that exocytotic and anti-oxidant mechanisms become impaired with age in the rat LG. In parallel with these structural alterations, functional declines may contribute to the pathophysiology caused by tear film modification in dry eye disease.

## Introduction

During aging, some harmful chemicals accumulate that can disrupt cell and tissue function. Such pathophysiological changes have been suggested to increase the possibility of illness and premature death [1]. The visual system is one of those most affected by aging. Clinical and experimental studies have shown that aging impairs tear secretion and induces changes in the lacrimal gland (LG) function and ocular surface properties [2]. However, there is limited understanding of the underlying mechanisms accounting for these changes.

In humans, the aging LG is at an increased risk of periductal fibrosis, infiltrated atrophy to acinar cells, and inflammation [3]. As shown in a rat experimental aging model, after 12 months, acinar cells synthesize less lacrimal film proteins, such as lipocalin, lysozyme, peroxidase, lactoferrin, betalisin, and immunoglobulin [4]. In parallel, there is an impairment of metabolic and neurogenic processes, which are critical for the function of several organs [5,6]. Other studies identified declines in insulin secretion and parasympathetic signaling, in parallel with an increase in hormone resistance and the accumulation of advanced glycation end products in the aging LG [7-9]. Moreover, those signaling pathway changes associated with age-related increases in oxidative stress have been detected in aging LG and are thought to contribute to tear dysfunction and dry eye syndrome [8,10].

The increases in age-related LG oxidative stress also stem from declines in oxidative stress scavengers and defenders, in addition to falls in peroxidase [8]. Those agents include enzymatic and non-enzymatic anti-oxidants, such as beta carotene, vitamin C, vitamin E, and glutathione [11].The liver plays a key regulatory role in storage and metabolism of anti-oxidants for the whole body. However, their activities are not affected by aging [12]. Among those anti-oxidants, vitamin E is of special interest because it is available to be administered systemically with therapeutic purpose and is being advocated for treatment of dry eye secondary to DM and also for age-related diseases [13,14].

Another predictor of age-related LG changes is the induction of exocytotic defects. This is evident since in non-obese diabetic mice (NOD), and Sjogren’s syndrome, changes occurred in secretory vesicles and exocytotic pathways as early as the first month. As a consequence, exocrine glands and tear function became impaired with age [5,15,16].

Exocytosis of the components of tears from the LG depends on a mechanism that is tightly controlled by local and systemic receptor-mediated signaling pathways. They include responses to cholinergic and adrenergic agonists that involve cAMP-mediated entry of calcium in acinar cells [17-19]. Intracellular vesicular transport and exocytosis are regulated by cytosolic protein families, called Rab GTPases and receptors of synaptic vesicles (SNAREs). The former are responsible for tethering vesicles to the target site, whereas in neural, endocrine, and exocrine tissues, the latter ensures their secretion across plasma membranes [20-23].

The SNARE and Rab proteins recently described in LG include vesicle-associated membrane protein-2 (Vamp-2), Ras related in brain GTPase protein-3 (Rab-3), and syntaxin. They are responsible for the docking of molecules and driving the fusion of vesicles with the plasma membrane [16,24]. The vesicular protein contents are constitutively released into the tears.

As both anti-oxidative and exocytotic functions are essential for preserving LG homeostasis, impairment of SNARE and secretory activity by age-related increases in reactive oxygen species can lead to ocular surface disease [25,26]. However, it is unclear in the rat LG if age-related increases in oxidative stress byproducts underlie declines in vesicular secretion.

We describe here in the rat LG the association between age-related changes in expression levels of oxidative damage markers and Rab and SNARE family proteins. These alterations are correlated with LG secretory vesicle structural modifications and ocular surface changes.

## Methods

### Animal model

Two- and 24-month-old male Wistar rats obtained from the Animal Breeding Center of the Faculty of Medicine of Ribeirão Preto, SP, Brazil were used after approval by the university’s committee on animal experimentation. The procedures adhered to the Principles of Laboratory Animal Care (NIH publication no. 85–23).

Anesthesia with ketamine (5 mg/100 g b.w.; União Química Faramacêutica S.A, Embu-Guaçu, SP, Brazil) and xylazine (2 mg/100 g b.w.; Laboratório Callier S.A., Barcelona, Spain) was used for comparative studies between both groups.

Bodyweight was recorded and tear secretion was measured in the right eye of rats in both groups using a modified Schirmer test (Ophthalmos, São Paulo, SP, Brazil), with a 1-mm width and 20-mm-long strip of filter paper placed in the conjunctival fornix of the eye for 5 min [27].

### Impression cytology

Cornea epithelial cells were collected from the temporal area with 45-μm filter paper (Millipore, Billerica, MA), fixed with 70% ethanol glacial ascetic acid 70% and formalin, stained with periodic acid-Schiff (PAS) and hematoxylin, and then transferred to microscope slides (n=11 in control group and 12 in aging group). Squamous metaplasia of epithelial cells was categorized in a masked fashion according to a four-stage classification scheme from 0 (normal morphology) to 3 (squamous metaplasia). The grading is based on the appearance of cytoplasm, presence and size of nuclei, as follows: stage 0 (for normal cell number, round morphology, and mucous staining), stage 1 (lower cell number and mucous staining), stage 2 (lower cell number, reduced size of nuclei, square shape of cells), and stage 3 (squamous metaplasia, showing lower cell number, higher cytoplasmic volume, and pycnotic or absent nuclei) [28]. The images were analyzed photographic documentation was done using a light microscope (Olympus BX40; Olympus Corporation, Tokyo, Japan) and a digital camera (Olympus Q-color 5; Olympus Corporation).

### Tissue collection and storage

Livers and LG collected (n=5/group) were homogenized for biochemical and western blot analysis. RNA from LG (n=5/group) were extracted by Trizol after homogenization (Invitrogen, San Diego, CA), and stored at −80 °C for later use.

LG samples for histology were collected, sectioned in the middle, and half were frozen in Optimal Cutting Temperature (OCT) compound (Sakura Fine Tek Inc., Torrance, CA), and the other half of the samples were fixed in 2% glutaraldehyde and 2% paraformaldehyde (EM Sciences, Hatfield, PA) in 0.1 M phosphate buffer, pH 7.4, for 40 min at room temperature (RT), for transmission electron microscopy (TEM).

### LG histology

OCT-embedded slides containing the 10th to the 14th sections of the LG of both groups were submitted for hematoxylin/eosin (H&E) staining (5 samples per animal, n=5/group). Digital photos were obtained from the H&E-stained and non-stained slides (for autofluorescence; Nikon Eclipse E800; Nikon USA, Melville, NY).

### Transmission electron microscopy (TEM)

LG tissues (n=5/group) fixed for EM were rinsed in 0.1 M phosphate buffer, dehydrated through a graded ethanol series, rinsed in acetone, and embedded in Embed 812 (EM Sciences). Sections (60–70 nm) were cut with a diamond knife and stained for 25 min each in 2% uranyl acetate and 5 min in Reynolds’ lead citrate. Sections were examined with EM (Jem 100cx; Jeol, Tokyo, Japan). Pictures were taken and converted to digital files (ORCA-HR Amtv542; Hamamatsu, Hamamatsu City, Japan).

### Biochemical analysis of oxidative stress and anti-oxidant markers

Malonaldehyde (MDA) was measured using the thiobarbituric acid-reactive substances (TBARS) in the LGs and livers of both groups [29]. Frozen LG and liver samples were homogenized in ice-cold 20% (w/vol) trichloroacetic acid, gently shaken for 30 min, and centrifuged at 5,000× g for 10 min. The supernatants (200 μl) were exposed to 0.7% thiobarbituric acid, heated to 95 °C for 45 min and, after cooling, absorbance was read at 530 nm in a Spectra Max 250 spectrophotometer against a blank sample (Molecular Devices, Sunnyvale, CA). TBARS concentration in the sample was calculated using a MDA calibration curve and expressed as mM/mg of tissue.

Reduced glutathione (GSH) levels in liver samples of both groups were determined as follows: frozen liver samples were homogenized in ice-cold phosphate (100 mM)-EDTA (1 mM) buffer (pH at 7.5) in a Potter tissue grinder with 4.0 ml of buffer for LG and liver, respectively. A 4.0-ml aliquot of the homogenate was removed and added to a tube containing 4.0 ml of deionized water and 1.0 ml of 50% trichloroacetic acid. After 15 min with occasional shaking, tubes were centrifuged at 3,000× g for 15 min at RT. A 2.0-ml aliquot of the supernatant was separated and 4.0 ml of 0.4 M Tris buffer, pH 8.9 and 0.1 ml of 0.01 DTNB in methanol were added to it. Optical density was measured at 412 nm 5 min later, against a blank solution with 0.02 M EDTA in place of the supernatant (DU 640; Beckman Coulter Inc., Brea, CA). Concentration was calculated using a standard GSH curve in EDTA (0.02 M). Data are expressed as nM/g of tissue [30].

Vitamin E was measured in LG of control and aged groups. Analysis was conducted by high-performance liquid chromatography (HPLC) with a column C-18 type (4,6 Shimpack CLCODSx 25 cm; Shimadzu Co. Kyoto, Japan), daily pay-column 4 mm×1 cm and 2.0 flow of ml/min [31]. Briefly, samples of LG were homogenized with 100% ethanol and hexane and then centrifuged for 15 min at 1,000× g. Aliquots of 1.0 ml were dried using nitrogen flow. The dried residue of each sample was resuspended in the mobile phase of acetonitrile/methanol/dichloromethane, submitted to HPLC, and read at 292 nm. Concentrations were calculated by comparison with standard samples of α-tocopherol and expressed in μM.

### RT–PCR for *Rab3d*, *Rab 27b*, and *Vamp-2*

*Rab3d*, *Rab 27b*, and *Vamp-2* mRNA expression levels were compared in the LG of both groups and β-actin (*Actb*) mRNA was used for internal normalization. The resulting RNA was quantified by measuring OD at 260 nm. RNA integrity was evaluated in 6.6% formaldehyde, 1% agarose (Gibco/BRL, Gaithersburg, MD) gels. Reverse transcriptase, oligo dT priming, and the Advantage RT-for-PCR kit from Clontech Laboratories Inc. (Palo Alto, CA) were used for cDNA transcription.

PCR amplification of cDNA was performed with a GeneAmp PCR System 9700 (Applied Biosystems, Foster City, CA) using 1.5 units of Taq DNA polymerase (Gibco/BRL), 0.3 mM each of dATP, dCTP, dGTP, and dTTP (Invitrogen), PCR buffer (Tris-Hcl 60 mM, MgCl_2_ 1.5 mM,NH_4_ 15 mM SO_4,_ pH 10; Invitrogen), and 10 mM of 5′ and 3′ primers (Life Technologies, Gaithersburg, MD) corresponding to rat *Rab3d*, *Rab 27b*, *Vamp-2*, and *Actb* cDNA (Table 1). Positive (pancreatic islets) and negative (without reverse transcriptase or cDNAs) controls were run in parallel.

**Table 1:** RT–PCR parameters for SNARE elements.

**Gene**	**Accession number**	**Primer sequence**	**Number of cycles**	**BP size**	**Annealing temperature**
*Actb*	NM_031144	Sense: 5′-agagggaaatcgtgcgtgaca-3′	33	202	59 °C
* *		Antisense: 5′-cgatagtgatgacctgaccgtca-3′			
*Rab3D*	NM_080580	sense: 5′-actgatggtgacaatgatgc-3′	37	340	59 °C
* *		antisense: 5′-acggaagtgaagaaagcaac-3′			
*Rab27b*	NM_053459	sense: 5′-cggagctcgagaagactaga-3′	37	225	60 °C
* *		antisense: 5′-ggccaggagtttaatcaggt-3′			
*Vamp2*	012663	Sense: 5′-gcatctctcctaccctttca-3′	34	141	58 °C
		Antisense: 5′-tttaggggtctgagggtaca-3′			

The PCR program used the following cycle profile: denaturation for 1 min at 94 °C, annealing for 1 min at indicated temperatures, extension for 1.5 min at 72 °C, and maximization of strand completion for 7 min at 72 °C. Following amplification, the cDNA fragments were analyzed on 1% agarose gels containing a 100-base pairs (bp) DNA molecular weight ladder (Gibco/BRL) and post-stained with ethidium bromide.

The results were resolved in Gel Doc (Bio-Rad Laboratories, Richmond, CA) and analyzed by Scion Image Analysis Software (Scion Corp, Frederick, MD).

### Western blot analysis

LG from both groups were solubilized in 1 ml buffer containing 100 mM 2-amino-2-hydroxymethyl-propane-1,3-diol (Tris; pH 7·5), 10 mM sodium pyrophosphate, 100 mM sodium fluoride, 10 mM EDTA, 10 mM sodium vanadate, 2 mM phenylmethylsulfonyl fluoride, and 1% Triton-X 100 and homogenized using a Polytron PT 1200C homogenizer (Brinkmann Instruments, Westbury, NY). The extracts were then centrifuged at 40,000× g at 4 °C for 5 min to remove insoluble material. Protein concentration in the supernatant fractions was assayed with the Bradford method [32]. The samples were treated with a Laemmli sample buffer and after heating at 95 °C for 5 min, the proteins were separated by SDS gel electrophoresis (100 μg protein/lane, 10% gels) and transferred to nitrocellulose membranes. The membranes were blocked with 5% non-fat dried milk, 10 mM-Tris, 150 mM-NaCl, and 0·02% Tween-20 overnight and were subsequently incubated with rabbit polyclonal anti-Rab3d, Vamp-2, Syntaxin, and GAPDH antibodies. GAPDH was used to validate protein loading equivalence (Table 2). Visualization of specific protein bands was made by incubating the membranes for 2 h with a peroxidase-conjugated secondary antibody (1:10,000; Zymed Laboratories, Inc., San Francisco, CA), followed by detection with enhanced chemiluminescence reagents (Pierce Biotechnology, Rockford, IL) and exposure to X-ray film (Kodak, Manaus, AM, Brazil). Band intensities were quantified by densitometry (Scion, Image, Frederick, MD).

**Table 2:** Western blotting antibodies used to compare SNARE expression levels in aging and control rat LG.

**Protein**	**Catalog number**	**Isotype**	**Molecular weight**	**Concentration**
GAPDH	Santa Cruz SC 25778	Rabbit polyclonal	37 kDa	200 μg/ml
Rab 3D	Santa Cruz SC 26392	Goat polyclonal	25 kDa	200 μg/ml
Syntaxin 1A	Santa Cruz SC 12736	Mouse polyclonal	35 kDa	200 μg/ml
Vamp 2	Calbiochem ≠ 627724	Rabbit polyclonal	12 kDa	1 μg/ml

### Statistical analysis

Data are reported as mean±SEM. Comparisons were made using the Mann–Whitney *U* test for continuous data and the Fisher exact test for categorical data and the level of significance was set at p<0.05 (GraphPad 5.0 software; Prism, San Diego, CA). Densitometry values are reported as a ratio of *Actb* in RT–PCR and GAPDH in western blot assays, respectively. The ratio of densitometric values of one control sample of each blot was defined as 1.0 (100%), and the subsequent values were expressed as a ratio relative to its control value and submitted to statistical analysis.

## Results

Body and LG weight were significantly higher in the aging group, as previously reported in this rat strain [9] (Table 3).

**Table 3:** Differences in structural LG and liver parameters control (2 months) and aged (24 months) rats (data are expressed as mean ± standard error).

**Parameter**	**2 months**	**24 months**	**p value**
Body Weight (g) *	272.0±11.6	551.7± 62.1	0.0043
LG Weight (mg) *	108.6±11.4	166.2±10.3	0.0079
LG Weight/Body Weight ratio (mg/g)	40.2±4.4	29.8±1.9	0.2222
Liver Weight (mg)	502.8±0.9	738.7±0.9	0.2468

* p<0.05, Mann–Whitney U test.

A modified Schirmer test showed that tear secretion did not decline during aging since it was 8.8±1.0 mm in the control group and 7.3±1.4 mm in the aging group (p=0.5145, Mann–Whitney U).

**Figure 1:**
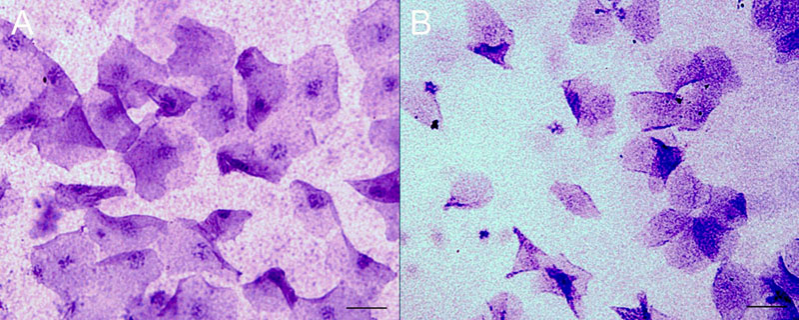
Impression cytology of the corneas of control (**A**) and aged (**B**) rats. Grades from 0 to 3 were given for each sample, based on size, nucleus, and the presence of mucus (Scale bar=25 µm).

Impression cytology (IC) to evaluate aging changes in the epithelial layer of the cornea presented a significantly higher frequency of alterations of the epithelial cells, with metaplastic keratinization in the aging group, in contrast with the control group, which presented samples of corneal epithelia with bigger nuclei area and round borders (p=0.032, Fisher test; Table 4 and Figure 1).

**Table 4:** Impression cytology of corneal epithelial cells of aged (24 months) and control (2 months) rats.

**Classification**	**2 month**	**24 month**
Grade 0	3	0
Grade 1	6	3
Grade 2	1	7
Grade 3	1	2

Cells were harvested with filter paper, transferred to slides, and stained with PAS. They were graded in a masked fashion according to a four-stage classification scheme from 0 (normal morphology) to 3 (squamous metaplasia), based on the color and format of the cytoplasm, presence and size of nuclei, and presence of mucous secretion (* p=0.032, Fisher Test).

**Figure 2:**
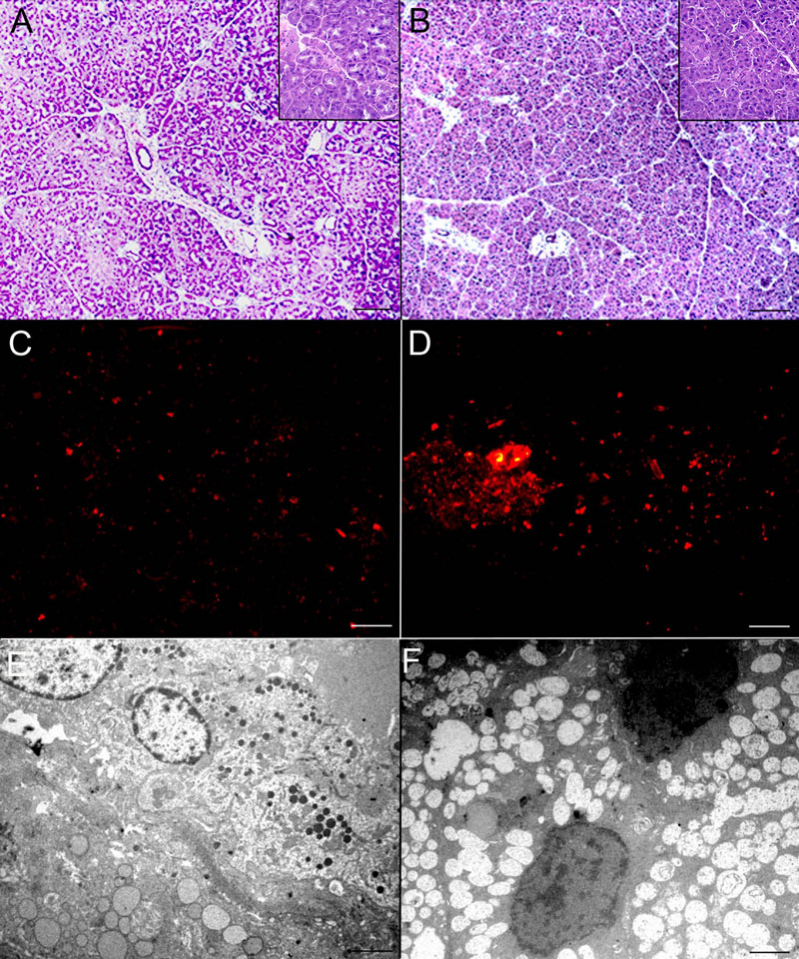
LG histology in the control and aged groups. Control (**A**) and aged (**B**) samples stained with H&E (Scale bar=100 µm). Unstained samples of control (**C**) and aged (**D**), to demonstrate autofluorescence of LG (white arrows: lipofucsin-like deposits; Scale bar=100 µm). TEM (Scale bar=2.5 µm) of control (**E**) and aged (**F**) samples revealing details of LG acinar cells.

H&E staining to compare the morphology of aging and control LG revealed similar acinar and ductal structures in both groups (Figure 2A,B). Autofluorescence indicated greater accumulation of lipofucsin-like material, which is a marker of age-related oxidative damage, in histological samples of the aging group LG compared to controls (Figure 2C,D).

Transmission electron microscopy, used here to compare details of cytoplasmic and nuclear structure of LG acinar cells between aging and control rats, revealed a reduced number of structured organelles, possible autophagic vacuoles, but an otherwise normal nuclear appearance without chromatin condensation in LG acinar cells of the aging group. In contrast, there were abundant secretory granules of diverse sizes and well structured organelles in the control group, suggesting preserved secretory machinery (Figure 2E,F).

Since oxidative stress has been implicated in the pathogenesis of dry eye in the elderly, our study compared the levels or oxidant and anti-oxidant markers in LG of aging and young rats. MDA, a marker of lipid peroxidation and oxidative stress, was similar in the control and aged groups. In contrast, in the aged group, vitamin E levels, indicators of anti-oxidant capacity, were lower in LG, suggesting a lower capacity of oxidative stress defense (Table 5).

**Table 5:** Comparison between the biochemical parameters of the LG and liver of the control (2 months) and (24 months) aged rats.

**Parameter**	**2 months**	**24 months**	**p**
LG MDA µM/mg tissue	11.0±0.9	24.6±1.7	0.0571
LG Vitamin E μM*	4.9±0.5	1.9±0.2	0.0159
Liver GSH µM/g tissue	4.8±0.4	3.5±0.8	0.2468
Liver MDA µM/mg tissue	240 ± 28	200 ± 30	0.6623

Data expressed on mean±standard error. * p<0.05.

**Figure 3:**
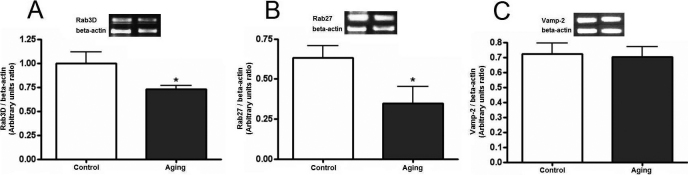
Effect of the aging on mRNA expression in LG. The ratio between *Rab 3d*/*Actb* (**A**), *Rab 27*/*Actb* (**B**), and *Vamp-2*/*Actb* (**C**) mRNAs are expressed as the mean±standard error of densitometric arbitrary units (*p<0.05, Mann–Whitney U test). Results are representative of three independent experiments.

Considering that Rab and SNARE are major cytoplasmic proteins involved in vesicular transport and exocytosis, the impact of aging on the LG secretory machinery was evaluated based on changes in gene and protein level expression of some mediators of this response. The ones chosen were shown to be affected by oxidative damage and are impaired in animal models of dry eye [24,26]. RT–PCR results revealed that *Rab3d* and *Rab27b* mRNA expression was 25 and 40%, respectively, lower in LG of the aging group, compared to *Actb*; however, *Vamp-2* was similar in both groups (Figure 3).

**Figure 4:**
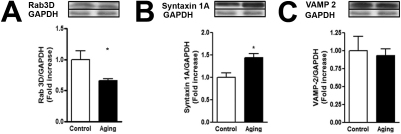
Effect of aging on the expression of the ratio of (**A**) Rab 3d/GAPDH, (**B**) Syntaxin 1A/GAPDH, and (**C**) Vamp 2/GAPDH in LG. Tissues from both groups were excised and homogenates were analyzed by western blot (*p<0.05, Mann–Whitney U test). Results are representative of three independent experiments.

Western blot analysis of LG whole cell lysates revealed disparate changes in Rab3d, Syntaxin, and Vamp-2 expression levels. In the aging group, Rab3d declined by 34% (p=0.008), whereas Syntaxin increased by 44% (p=0.0159). On the other hand, Vamp-2 expression did not change (p=0.69; Figure 4). Together, those data suggest that aging changes the expression of proteins related to exocytosis and biomarkers of oxidative stress in LG, and these may regulate the expression of their downstream signaling connectors, affecting lacrimal secretion.

## Discussion

The present work extends previous findings of structural and biochemical changes in LG aging [8,9]. Aging affects secretory and anti-oxidant mechanisms, which are two major functions for proper physiology and homeostasis of LG. These changes implicate extended exposure to oxidative stress as a possible cause for a reduction in the lacrimal gland secretory function. Such a decline may play a role in development of age-related dry eye [5].

Unlike previous findings, we could not detect that aging results in a fall in tear secretion and a 60% decline in protein content. Our failure to identify a decline in tear secretion could be due to a difference in methodology. We used a modified Schirmer test, which is probably less sensitive than using tears collected from the conjunctival fornix to measure their protein content [8,33].

Markers of oxidative stress increased based on biochemical and histological analyses from aging rats. Moreover, levels of the anti-oxidant alpha-tocopherol fell in parallel, suggesting that a reduced anti-oxidant capacity contributes to aging dysfunction. If such a change also occurs in dry eye patient tears, it may contribute to the elevations in tear lipid peroxide levels and increases in ocular surface damage measured in their tears [34]. This suggestion is supported by our finding of higher levels of MDA and lipofucsin in LG of aging rats. On the other hand, in the same aging rats as those used for assessing changes in LG function, their liver levels of oxidative stress markers were unchanged. This invariance is in agreement with previous publications [12,35].

Aging had no impact on LG histology. Furthermore, unlike in humans and mice, but in agreement with previous studies in rats, there was no obvious inflammatory infiltration into the LG [3,7,36]. However, the marked appearance of autofluorescence that we detected was reported in aging mice and diabetic rats, suggesting a correlation between metabolic impairment, lipofucsin accumulation, and functional disruption [7,9,37].

Our results showed that declines in tear anti-oxidant capacity with aging were associated with alterations in corneal epithelial impression cytology. This association has also been observed previously in dry eye models related to hypothyroidism [27]. Similarly, higher levels of pro-inflammatory or oxidant mediators could explain the higher frequency of corneal metaplasia and declines in epithelial turnover [8,34]. Such changes that we identified may also be explained by declines in corneal innervation and deterioration of limbal cell function [38,39]. Another possible outcome of aging is its correlation with declines in K10 cytokeratin expression, which is associated with increases in proliferation and migration of corneal epithelial cells. Such changes could be associated with higher levels of corneal epithelia metaplasia [40]. Therefore, declines in anti-oxidant capacity and increases in pro-inflammatory mediators may have numerous effects associated with suppression of corneal epithelial function and turnover.

The presence in aging LG of lipofucsin-like bodies in autofluorescence and structures suggestive of autophagic vacuoles in acinar cells in combination with higher expression of syntaxin, a member of the SNARE family, would not appear to be suggestive of declines in LG function with age [41,42]. However, recent studies revealed that members of the SNARE family and, in particular syntaxin-5, are necessary for the clearance of autophagic products [43,44]. As autophagic vacuoles are the result of organelle degradation to remove lipofucsin-like products related to aging oxidative stress, the unchanged or increased levels of members of the Rab and SNARE family may have adaptive value in supporting removal of those ROS byproducts [45,46]. Another possible explanation for differences in aging LG between Rab and SNARE expression levels is that it is a compensatory response in the later signaling steps (i.e.; Vamp and Syntaxin) to declines of Rabs, secretory vesicles, and secretory activity. Further studies focused on details of secretory pathways are necessary to test these hypotheses.

Caloric restriction (CR) is the only known mechanism that modulates the aging process [47]. This finding is consistent with a report on the LG, in which a decline in caloric intake of only 35%, initiated in adult life, reduced the impact of aging on LG function [48]. Such an observation has potential clinic relevance, but reducing caloric intake should exclude a fall in vitamin intake; otherwise, it could affect several organs, including decreases in exocrine function [14,49,50].

Further studies are needed to determine whether vitamin E supplementation can offset declines in LG function during aging even though it is currently advocated therapeutically for certain conditions including dry eye [13].

In conclusion, our data show that some of the anti-oxidant and secretory mediators’ expression levels become impaired in the aging LG. Although the underlying mechanisms for these changes leading in some cases to dry eye are not fully clarified, some of the protective mediators that would appear not to be supportive of LG function were identified. Such insight may lead to the identification of novel strategies that protect or at least delay the onset of age-related LG dysfunction.
